# Colloidal Stabilization of Submicron-Sized Zeolite NaA in Ethanol–Water Mixtures for Nanostructuring into Thin Films and Nanofibers

**DOI:** 10.1021/acs.langmuir.2c02241

**Published:** 2022-12-20

**Authors:** Oğuz Gözcü, H. Utkucan Kayacı, Yibo Dou, Wenjing Zhang, Niklas Hedin, Alma B. Jasso-Salcedo, Andreas Kaiser, Simge Çınar Aygün

**Affiliations:** † Department of Metallurgical and Materials Engineering, Middle East Technical University (METU), 06800 Ankara, Türkiye; ‡ Department of Environmental Engineering, Technical University of Denmark, Bygninstorvet, 2800 Kongens Lyngby, Denmark; § Department of Energy Conversion and Storage, Technical University of Denmark, Anker Angelundsvej, 2800 Kongens Lyngby, Denmark; ∥ Department of Materials and Environmental Chemistry (MMK), Stockholm University, Svante Arrhenius väg 16 C, 10691 Stockholm, Sweden

## Abstract

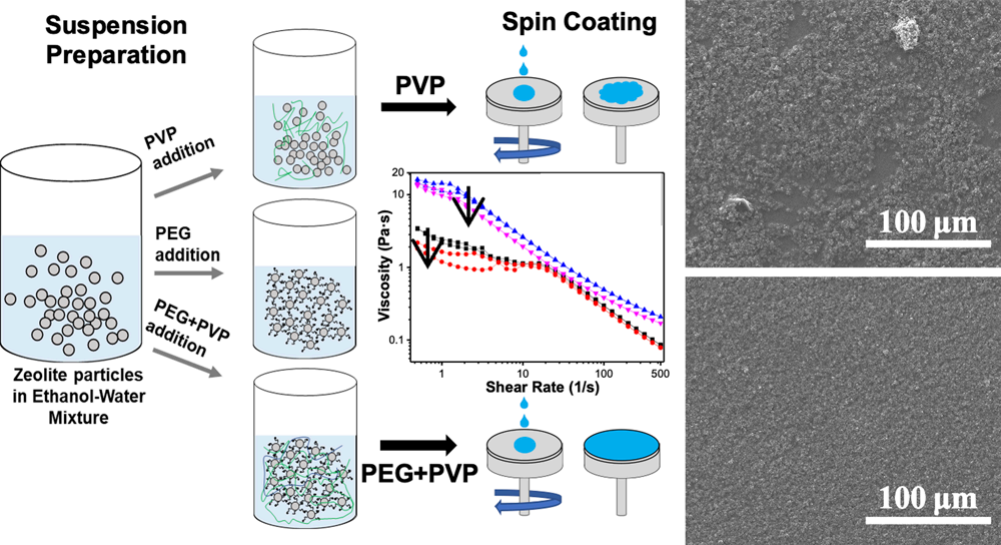

Despite the growing use of organic or mixed solvents in zeolite processing, most studies focus only on aqueous suspension systems. We investigated the colloidal characteristics of submicron-sized zeolite NaA in mixed ethanol–water solvents. The effects of the mixing ratio of solvents and various additives on the dispersion of the zeolite powders were studied. The zeolite NaA particles were destabilized in solvent mixtures at a high ethanol-to-water ratio, a reduction in the zeta potential was observed, and the destabilization was rationalized by the Derjaguin, Landau, Verwey, Overbeek (DLVO) theory. An improved stabilization of the zeolite NaA suspensions was achieved in ethanol-rich solvent mixtures using nonionic low molecular weight organic additives, but not with their ionic counterparts such as anionic, cationic surfactants or inorganic acids or bases. Polyethylene glycol (PEG)-400 was found to be a good dispersant for the submicron-sized zeolite NaA particles in the ethanol–water mixtures, which was attributed to its interaction with the zeolite surface, leading to an increased zeta potential. The PEG-stabilized zeolite suspensions led to low suspension viscosities as well as uniform and consistent spin-coated films.

## Introduction

Zeolites are microporous crystalline aluminosilicate materials^1−3^ with framework type codes (FTC)^4,5^ and are used as adsorbents,^6^ catalysts,^7,8^ ion exchange materials,^9^ and molecular sieves.^10,11^ They are continuously being explored also for other applications including in nanotechnology,^12,13^ membrane technology,^14,15^ microelectronics,^16^ biotechnology,^17^ CO_2_ capture and storage,^18^ water treatment, etc.^19^

As zeolite powders cannot be used as such, they are shaped into functional forms. Various shaping approaches are explored, such as *in situ* growth,^20,21^ seeded growth,^22^ gel-based deposition,^23^ slurry-based wet deposition,^24−28^ and complex shaping methods, such as electrospinning,^29−31^ additive manufacturing,^32−35^ etc. In the mentioned approaches, the colloidal zeolite suspensions are either deposited on substrates or used as an ink for the shaping. One example is the spin coating of zeolite structures for various applications. Mintova and Bein^36^ reported the direct formation of zeolite thin films (ZSM-5; FTC = MFI) from stable colloidal suspensions of zeolites, resulting in the formation of uniform microporous films on almost any substrate and with preferred orientation and tunable thicknesses.^37^ Huang et al.^26^ showed the scalable fabrication of zeolite thin films using direct wet deposition of colloidal zeolite Y suspensions (FTC = FAU, with Si:Al = 0.14). Using a similar direct wet deposition technique, Hsu et al.^28^ reported the preparation of a zeolite-like antifogging coating on a glass substrate. By spin coating the glass with colloidal suspensions of the microporous silicalite (FTC = MFI), they could avoid the commonly harsh environments used for zeolite crystal growth reactions. In that study, the importance of the colloidal stability of the zeolite suspension and the use of appropriate surfactants were emphasized. Similarly, Lam et al.^27^ reported on the deposition of zeolite films with ultrasonic nozzle spraying, in which the effects of the cast suspension’s compositions were optimized to obtain crack-free, continuous, and thin films with a homogeneous zeolite distribution and desired thickness. Another example for utilizing zeolite dispersions in advanced shaping techniques is additive manufacturing. Very complex structures of NaA zeolite structures have been realized by 3D printing to achieve high performance in gas separation including CO_2_ capture^38−40^ and water purification.^41,42^ Thakkar et al.^43^ studied the fabrication of 3D-printed monoliths of zeolites using a robocasting technique. To obtain the optimum suspension viscosity, zeolites were prepared in aqueous media with organic binders and plasticizers to enable the extrusion process, which were then removed in the calcination step.^32,38,44^ Following this study, the examples of the fabrication of complex-shaped zeolites using extrusion-based additive manufacturing techniques from zeolite suspensions have been varied. Improving the mechanical strength and increasing the loading of particles in such structures were the main aims of these studies. The rheological behavior and the suspension formulation were highlighted due to their importance not only for the shaping process but also for obtaining high-quality end products with a homogeneous distribution of zeolite powders.

Finally, electrospinning is an advanced shaping technique, which has been used to structure microporous framework materials either into 1D fiber structures or their more complex 3D variations for a broad range of applications, such as biomedical applications, air filtration, catalytic applications, water treatment and gas separation processes.^45−48^ As an example, in gas adsorption processes hierarchical porous zeolite nanofiber structures can reduce pressure drop and improve the diffusion of molecules.^30,49^ However, the zeolite particles are usually embedded in the nanofiber polymer matrix after electrospinning. It has been shown that the coverage of the zeolite surfaces with the polymer often leads to a significantly reduced surface area and accessible pore volume, resulting in significantly lower gas uptake. The removal of the polymer matrix by a subsequent heat treatment step (carbonization) has been proposed to recover the multimodal open pore structure of the zeolite particles in a zeolite-carbon composite structure. In the electrospinning process, the homogeneous distribution of powders in polymer solutions is desired for processability of electrospun fibers.

These examples emphasize that the structuring of zeolites in advanced shaping techniques^50−52^ has become of critical importance today. Considering this, the open literature provides surprisingly a very limited number of studies related to the colloidal properties and colloidal processing conditions of zeolite powders and is commonly limited to studies of electrostatic stabilization of zeolite powders in water-based solvents. For instance, in the report of Nikolakis,^50^ the interactions in zeolite colloidal suspensions were reviewed, with particular focus on zeta potential measurements and their relation with the particle nature. Kuzniatsova et al.^53^ reported the electrostatic stabilization of zeolite Y powders in aqueous media. Based on the zeta potential measurements, the optimum suspension conditions, thus the homogeneous deposition of particle coating, were obtained by adjusting suspension pH at low ionic strength. Oonkhanond and Mullins^51^ studied the relationship between the film-forming ability of the zeolite particles and the electric double layer effects. They applied the DLVO (Derjaguin, Landau, Verwey, and Overbeek) theory. This theory combines the attractive van der Waals interactions and repulsive electrostatic (double layer) interactions between particles in dispersion. The net calculation of the interaction potential between two particles^54^ informs of the tentative stability field of such particles. Based on these calculations, Oonkhanond and Mullins explained the difficulty in forming continuous zeolite A (FTC of LTA) films with the stronger repulsive interactions both between the zeolite A particles and between the zeolite A and the substrate as compared to the interactions in the case of zeolite ZSM-5 particles. Zeolite A has a silicon to aluminum ratio of one, while zeolite ZSM-5 has much less aluminum and hence much less charge. Akhtar and Bergström^55^ reported the colloidal processing of hierarchically porous zeolite 13X monoliths. The zeolite particles with a size between 3 and 5 μm were found to have an isoelectric point (IEP) at pH 4.7 and electrostatic stabilization was promoted by adjusting the pH to 9.6. The high negative zeta potential of the zeolite particles in alkaline media prevented the particles from agglomerating in aqueous media, leading to lower suspension viscosity and enabling the processing of stabilized suspensions without using binders. In another study, Akhtar et al. reported on a similar colloidal procedure to coat the walls of a foam-like microporous alumina support with zeolite 13X particles, and get benefit from the colloidal stability and the rheological properties of the aqueous zeolite suspensions, ensuring high-quality and homogeneous coatings.^56^ Liu et al. investigated the effects of internal structure, acidity, pH, temperature, and concentration of zeolites on their zeta potentials due to its importance on the preparation of metal-supported catalysts and shaping of zeolites into extrudates.^57^ In the study of Ogura et al.,^58^ it is stated that the changes in solution pH lead to changes in structural and acidic properties of zeolites. To the best of our knowledge, the effects of organic additives or the polymer-carriers in the stabilization of zeolite suspensions have not been discussed extensively in the open literature. Moreover, most of the colloidal properties of zeolite suspensions have been investigated in aqueous media. However, with the increasing interest in new processing and shaping techniques, the use of organic or mixed solvents is becoming important. These solvents have high volatility, low surface tension, and good ability to dissolve polymers.

Colloidal systems in mixed solvents are of interest for shaping zeolite powders into nanofibers via electrospinning^30^ or into nanocoatings via spin-coating,^59^ or in tape casting. With this work, we contribute to understanding of the colloidal stabilization of nanozeolite powders in complex solvent mixtures of water and alcohol, utilizing different type of organic additives and polymer-carriers. We investigated the effects of various types of additives on the stability of submicron sized zeolite NaA in different ethanol–water mixtures, including zeta potential measurements. Experimental results were rationalized in a DLVO theoretical framework. Finally, the zeolite suspensions were evaluated in terms of their rheological behavior and the resulting coating quality after a spin-coating process.

## Experimental Section

### Materials

Zeolite NaA powders, prepared by hydrothermal synthesis according to the procedure reported before,^60,61^ were used in the experiments. Si:Al and Na:Al ratios of the synthesized powders are 1.1 and 0.66, respectively (Table S2). The density of the powder was 2.0 g/cm^3^. Poly(vinylpyrrolidone) (PVP) with a molecular weight of 1 300 000 g/mol was purchased from Sigma-Aldrich. Polyethylene glycol (PEG-400, molecular weight between 380 and 420 g/mol, Zag Chemicals), low molecular weight PVP (avg molecular weight: 1500 g/mol, Sigma-Aldrich) as a nonionic additive, hexadecyltrimethylammonium bromide (CTAB, Sigma-Aldrich) as a cationic surfactant and sodium dodecyl sulfate (SDS, Sigma-Aldrich) and poly(acrylic acid) (PAA, Sigma-Aldrich, av. molecular weight: 5000 g/mol) as anionic additives were used as additives and HCl (purity >98%) and NaOH (purity > 98%) were used to adjust the pH and supplied from Sigma-Aldrich. Ultrapure water with a resistivity of 18.2 MΩ and a technical grade ethanol (purity ≥ 96%) were used for the preparation of the suspensions.

### Powder Preparation

The zeolite NaA was exposed to a relatively high energy process; either by ball milling or by ultrasonic treatment to break the agglomerated zeolite particles. In the case of ball milling, ultrapure cylinder-shaped zirconia (ZrO_2_) balls, 99.99% with 0.5 mm in diameter and 4.5 mm in height were used. The weight ratio of the liquid medium to the powder was 4:1 (wt) and powder to ball ratio was 1:10 (wt). In each set, powders were added into 50 g of a liquid medium (ultrapure water or ethanol) and ball milled at 50 rpm in a 100 mL bottle made of polyethylene terephthalate. To reduce the ball-milling time, powders were ball milled in a relatively higher energy ball-mill setup at 100 rpm (Fritsch Pulverisette 7). In this set, powders were added into 25 g of a liquid medium (ultrapure water) and ball-milled in 45 mL zirconium oxide vessels.

For the ultrasonic treatment, a suitable ultrasonic pin (sonotrode S7) was used in a liquid medium by connecting UP200 St ultrasonic lab device (Hielscher Ultrasonics GmbH). Three grams of powder were mixed in 20 g of liquid medium and exposed to an ultrasonic treatment at a power ranging from 35 to 100 W at 25 kHz. After either process, the powders were dried at 120 °C overnight.

### Suspension Preparation

For practical purposes, ultrasonicated powders (at 70 W for 5 min) were used in sedimentation experiments, while ball-milled ones (at 100 rpm for 24 h) were preferred to be used in rheological measurements because of the need for larger amounts.

For sedimentation experiments, first ethanol and ultrapure water were mixed in a 50:50 (wt %) ratio and shaken for 3 h at 80 rpm using a shaker (Isolab 3D orbital shaker). Then, zeolite powders were added to the solvent mixture at a concentration of 1.5 wt % for the sedimentation experiments, 10 wt % for the spin coating experiments, and 30 wt % for the rheological measurements. For sedimentation experiments, dilute suspensions were preferred to eliminate strong interparticle interaction. For spin coating experiments, 10 wt % of particle loading was found optimum for efficient coating. For rheological measurements, to amplify the effect of additive on the suspension viscosity, denser suspensions were used. The suspensions were ultrasonically treated for 3 min with a power of 35 W for homogenization. Cooling breaks of 15 s were introduced every minute to prevent overheating and vaporization of the solvent and to keep the solid loadings constant.

For additive (PAA, CTAB, SDS, PEG, or low molecular weight PVP) containing suspensions, the calculated amount of additive was first added to the ethanol–water (50:50 wt %) solution and then, the mixture was stirred for 2 h at 80 rpm for a complete dissolution of the additive. Then, zeolite powders were added into the solution and the suspension was ultrasonicated for 3 min at 35 W. For the suspensions with carrier polymer, high molecular weight PVP was added into the prepared suspensions and ultrasonication was employed.

### Characterization of the Zeolite NaA Powders and Suspensions

The morphology and the agglomeration state of the zeolite NaA powders were characterized by scanning electron microscopy (SEM, FEI Quanta 400F Nova NanoSEM 430 SEM System, Oregon, USA). To minimize the sample charging, the powders were coated by a thin layer of gold by a Polaron SC 7640 Sputter Coater (Watford, UK) for 2 min at the setting of 1.5 V and 10 mA. Energy Dispersive X-ray Spectroscopy (EDX, JEOL 2100F, Japan) was used at 20 kV for 90 s for elemental analysis of the zeolite powder. For the analysis, powders were dried at room temperature for about 12 h. The powder was mixed with ultrapure water and a drop from this suspension was taken to the carbon tape and left to dry prior to the EDX analysis. The ion ratio was measured by taking the average of at least three measurements.

The particle size distributions of the zeolite powders were examined using a laser diffraction technique (LD, either with Beckman Coulter LS 13 320 or Mastersizer 2000 with HydroMu dispersing unit, Malvern Instruments). The LD analysis was performed in ethanol, in water, or in their mixtures at room temperature (25°C). The results of LD measurements were reported as an average particle size based on the weighted averages of the scattered light intensity. Each LD analysis was repeated 3 times for each specimen under the same conditions, and the variations in the particle size measurements were not higher than 2%.

X-ray diffraction (XRD) analyses were performed using an X-ray diffractometer (Bruker D8 Advance) with Cu Kα irradiation (0.154 nm) at a working voltage of 40 kV and a scanning speed of 2° min^–1^.

CO_2_ adsorption isotherms were recorded in a Micromeritics ASAP2020 analyzer. The samples were dried at 80 °C overnight to remove moisture and solvent residue. The samples were subjected to dynamic vacuum at 623 K for 5–10 h prior the gas adsorption experiments. The CO_2_ adsorption experiments were performed at 273 K and the temperature for the sorption experiment was kept constant using ice bath.

Zeta potential measurements were conducted using a Zetasizer Ultra (Malvern Instruments) at 25 °C. For the analysis, suspensions of the zeolite powders were prepared with a concentration of 0.1 wt %.

For pH measurements, a standard pH-meter was used, and the measurements were carried out 2 h after suspension preparation. The reported pH values do not reflect the absolute proton concentration in solution, but it corresponds to an equivalent value in the ethanol–water mixtures.

The chemical interactions between PEG, PVP, zeolites, and solvents (ethanol and or water) were analyzed using an infrared (IR) spectrometer with an attenuated total reflection (ATR) accessory (PerkinElmer). The Spectrum 10 software was used to analyze the spectra. Before the examinations of the spectrum, interactive baseline correction at 4000 cm^–1^ was applied. The related spectrum was subtracted from each other to amplify the difference between the spectra. For example, to analyze the interactions between the PVP molecules and zeolite-A powder, spectra of the zeolite-A suspensions were subtracted from the spectra of suspension containing PVP and zeolite-A. All IR analyses were conducted at room temperature (25 °C) and recorded over the wavenumber range of 4000 to 400 cm^–1^.

To measure the sedimentation rate of the zeolite powders, the settling rate of the particles was observed via the amount of powders accumulated at the bottom of a 10 mL cylinder. Here, 1.5 wt % of the zeolite powder was slowly added into the solvent of interest. All sedimentation measurements were conducted at room temperature.

The rheological behavior of suspensions was analyzed using a rheometer (MCR 102, Anton Paar). A cone-and-plate geometry (a steel cone with an angle of 4° and 40 mm in diameter) was used as an attachment for the measurements. A solvent trap was used to prevent evaporation from the water–ethanol mixture. The shear rate was initially increased from 0.5 to 500 s^–1^ and then decreased back to 0.5 s^–1^. For data precision, three consecutive cycles were recorded with 31 points of each half loop at 25° ± 0.1 °C. The data were reproducible after the first half loop and the third run’s loop data are presented.

### Spin-Coating

Silicon wafers (0.5 × 1 cm^2^) were used for spin-coating. They were first immersed in acetone for 3 min, and then placed in ethanol for another 2 min for cleaning. Subsequently, they were dried using pressurized air at room temperature. The zeolite films were prepared by using a commercial Spin-Coater KW-4A (Chemat Technology Inc., Northridge, CA). A volume of 100 μL of a zeolite suspension including 10 wt % powder was dropped at the center of the substrates under stationary conditions. Then, the spin coater was accelerated to 3000 rpm within 3 s and rotated at this speed for 45 s. The zeolite films were dried at room temperature and characterized with SEM.

### DLVO Theory Calculations

**Figure 1:**
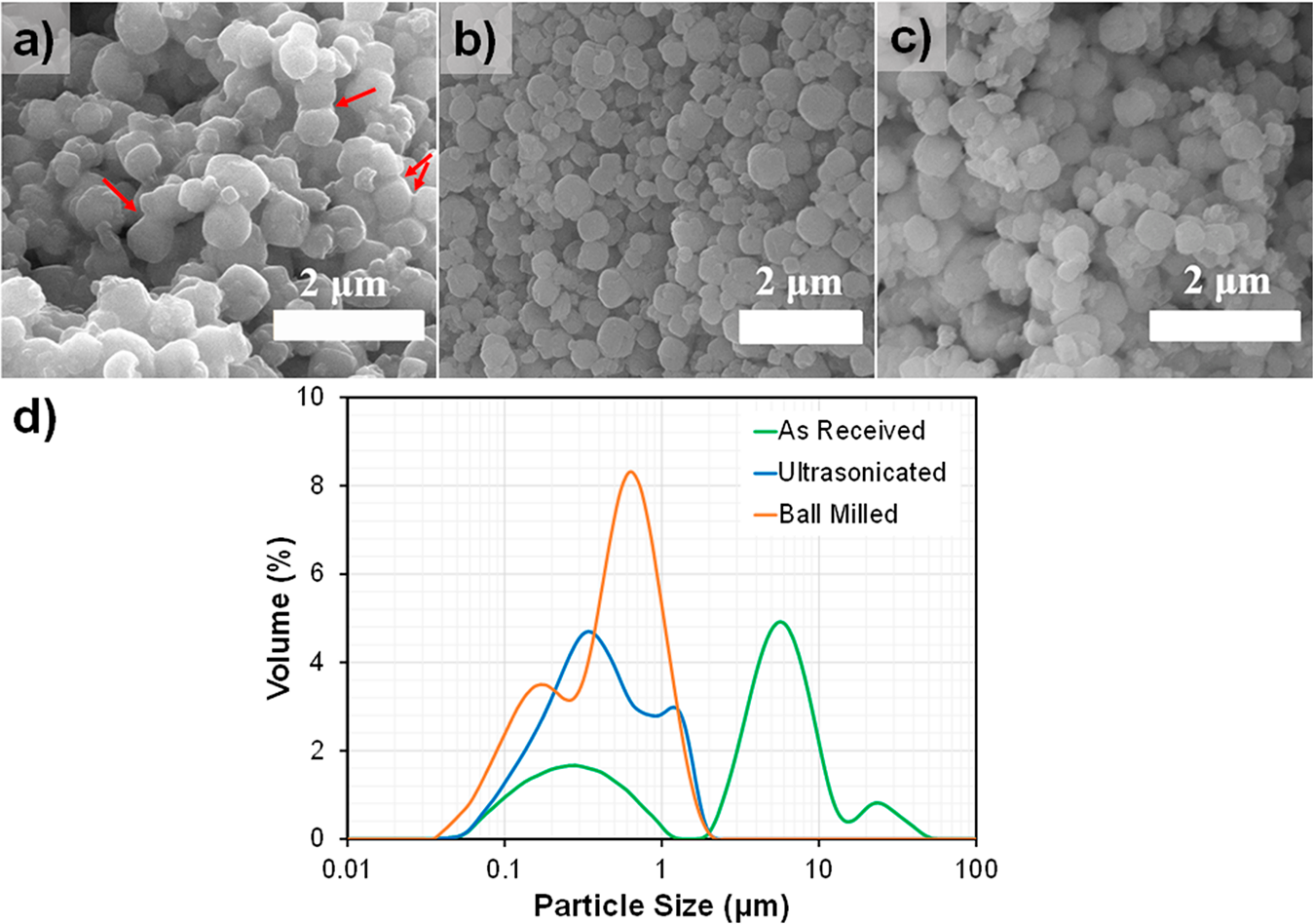
Physical characterization of as-synthesized and treated zeolite |Na_12_|-A powders. SEM micrographs of the as-synthesized/received (samples were shipped from Sweden) powder (a); powders after ball milling at 100 rpm for 24 h in water (b); and powders after ultrasound treatment at 70W for 5 min in water (c). Red arrows in (a) show the necking between particles. Particle size distribution of the zeolite NaA as-synthesized and as-treated powders in water (d).

For DLVO theory calculations, the Hamaker 2.2.2 software^62^ was used. The ion concentration was taken as 0.001 M and the temperature as 298 K. The particles were assumed to have monomodal distribution and have diameter of 300 nm, which is the average primary particle size obtained from experimental measurements (Figure 1d). Dielectric constants of ethanol–water mixtures were taken from study of Wyman.^63^ Hamaker constant was taken as 1.5 × 10^–20^ J from Oonkhanond and Mullins.^51^

## Results and Discussion

### Pretreatment of Zeolite Powders

Particle agglomeration is a common issue in the synthesis and application of nanopowders.^64^ Drying or sintering steps may lead to the formation of permanent agglomerates, which are difficult to redisperse, thus adversely affecting colloidal stability. To obtain high-quality and complex-shaped zeolites from suspensions, the zeolite powders should be homogeneously dispersed prior to processing. As shown in the SEM micrographs in Figure 1a, the zeolite NaA powders are composed of sphere-like individual particles with average particle size of about 0.3 μm. However, as inferred from the necking between particles, these primary particles are in the form of large agglomerates. In line with the SEM micrographs, the particle-size analysis presented in Figure 1d also shows that the as-synthesized zeolite powders exhibit a trimodal distribution of micron-sized agglomerates (∼23 and ∼5.6 μm) and smaller primary particles (∼0.3 μm). We hypothesized that stable suspensions of homogeneously dispersed powders can be obtained when the large agglomerates are broken into smaller particles (<1 μm), preferably to the size of the primary particles.

To mechanically break the agglomerates, ultrasonication and ball milling were applied. Once the process parameters were optimized (Figures S1 and S2), the necks between the primary zeolite powders were successfully broken up (Figure 1b–d), showing that the necks between the particles were not permanent. In the case of ball milling (Figure S1), the agglomerate size could be reduced when ethanol was used as a media; however, the portion of the secondary particles remained. With water as a milling media, primary particles with homogeneous size distribution could be obtained after 72 h of ball milling. To reduce the milling time, the milling rate was increased to 100 rpm, and all large agglomerates were successfully eliminated after 24 h of milling. It was found that 5 min of ultrasonication at a power of 70W was sufficient to eliminate agglomerates (Figure S2). At the end of either treatment, the particles were dried and redispersed for the particle size measurements, showing that the necks between the particles were not reformed. The pH of the aqueous zeolite suspensions was measured and no significant change was observed when compared to its initial value (∼10.3), indicating that the chemical properties of the powders were preserved at a certain level. The XRD diffractograms of powders (Figure S3) did not change after either treatment. Moreover, CO_2_ adsorption isotherms (Figure S4) showed only very minor deviations, indicating that there is no change in the crystallinity of the zeolite due to the ball milling or ultrasonication treatment.

### Effect of Ethanol Amount on the Stability of Zeolite NaA Suspensions

**Figure 2:**
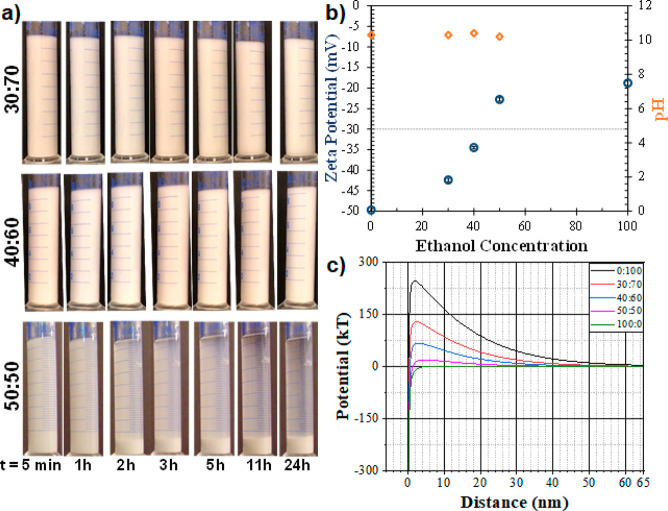
Stability of zeolite NaA particles in mixed solvents. (a) Sedimentation behavior of zeolite powder in mixed solutions. Ethanol:water ratios are 30:70 (top line), 40:60 (middle line), and 50:50 (bottom line). The images were taken in various time intervals as labeled at the bottom. (b) Zeta potential and pH measurements of zeolite-A in a mixed ethanol–water solvent. The plot was drawn with respect to the weight percentage of ethanol. (c) Results of DLVO theory calculations for zeolite NaA particles in mixed ethanol–water solvents. In legend, the ethanol:water ratio is presented.

While the powders were dispersible enough to conduct particle-size measurements in water, adding ethanol into the solution reduced the stability of particles and led to faster sedimentation. To assess the effect of the ethanol concentration on the dispersion quality of the powders, the sedimentation characteristics of the treated zeolite powders were studied. As shown in Figure 2a, the settling velocity of the zeolite NaA increased with an increasing amount of ethanol. The zeolite powders remained suspended in the mixed solution containing 30% and 40% ethanol for at least 24 h. Nevertheless, when the ethanol amount was raised to 50%, a significant amount of zeolite powders had settled in 3 h, indicating higher sedimentation rates as a result of the agglomeration of primary particles.

The accelerated sedimentation of the zeolite particles with an increasing ethanol amount in the ethanol–water solvent can be explained by the zeta potential values of the powders measured in the mixed solvents (Figure 2b). While the zeta potential value of the powder was as low as −49.7 mV in pure ultrapure water (0:100), it was only −18.8 mV in pure ethanol (100:0). The zeta potential values increased to −42.4, −35.4, and −22.8 mV when the ethanol concentration increased to 30, 40, and 50 wt %, respectively. It is known that zeta potential values greater than ±30 mV introduce high enough electrostatic repulsion between particles to typically stabilize dispersions or suspensions toward agglomeration.^65−68^ Therefore, zeolite powders in suspensions with ethanol amounts of 40 wt % or lower are expected to be electrostatically dispersed based on their zeta potential values. At higher ethanol concentrations, the zeta potentials were below this critical value, indicating an inadequate level of electrostatic repulsion between the zeolite particles and an increased tendency for agglomeration.

The total attractive and repulsive interaction potentials were assessed via calculations using the DLVO theory. As shown in Figure 2c, at ethanol concentrations of 50%, the repulsive energy barrier between particles is reduced significantly. The changes in surface charge density with increased ethanol concentrations result in lower zeta potential values and lower repulsive energy barriers.^69−71^ The changes are due to the surface-ethanol/water interactions and differences in dissociation constants of electrolytes because of the lower dielectric constant of ethanol (24.5) compared to water (78.1). As a result, when the particles get closer to each other in suspension, they will tend to agglomerate. With the formation of these secondary structures, i.e., agglomerates, the effective particle size will increase and sedimentation will occur as the particles will not resist the gravitational forces. On the other hand, when the ethanol concentration was <40 wt %, the natural surface charge, thus effective electrostatic repulsive interactions, was large enough to stabilize the zeolite NaA particles without any need for additional effort. Exactly the same cutoff value was seen both in the sedimentation results and in the zeta potential measurements. At higher ethanol concentrations, the zeolite NaA particles would tend to agglomerate because of the lack of a repulsive energy barrier induced by the electric double layer. In conclusion, additives were required to stabilize zeolite NaA particles in solvents with high ethanol content.

### Effect of PVP on the Stability of Zeolite Suspensions for Zeolite NaA

**Figure 3:**
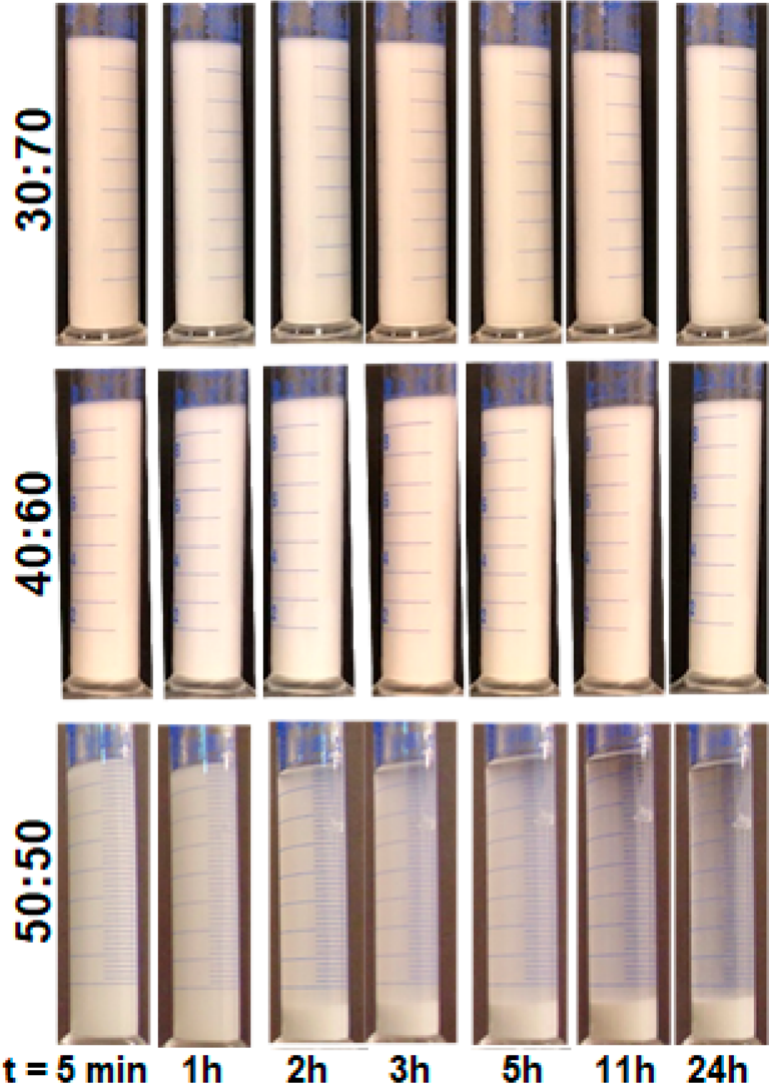
Sedimentation behavior of zeolite NaA powders in mixed ethanol–water solvents with addition of high molecular weight polyvinylpyrrolidone (1 300 000 g/mol). Ethanol:water ratios are presented on the left side of the images.

In many shaping processes, high molecular weight polymers are required to adjust the suspension viscosity and act as a binder or as a carrier. In such processes, a uniform distribution of zeolite particles in the polymer is vital for obtaining end products with uniform properties. These polymer-carriers are bulky units (on a microscopic level) and may expedite powder sedimentation. Here, we studied the sedimentation behavior of zeolite NaA with a commonly used polymer-carrier (high molecular weight PVP). As seen in Figure 3, even with PVP, the zeolites in suspension (containing 30 and 40 wt % ethanol) did not sediment before at least 24 h. However, significant sedimentation was observed even after an hour when the ethanol concentration was 50 wt %.

Zeta potential measurements revealed that the stability of the zeolite NaA suspensions was reduced slightly in the presence of PVP (Table 1). The decreases in zeta potential values were minor when the water content was high. However, in the case when ethanol concentration was increased to 50:50 wt %, significantly higher zeta potential values (less negative) were recorded for the suspension with PVP. Lower zeta potential values indicated specific interactions between the zeolite particles and PVP.

**Table 1:** Zeta Potential Values of Zeolite NaA in Ethanol–Water Solutions and the Natural pH of their Suspensions with or without Polyvinyl Pyrrolidone (PVP) Addition

	zeolite NaA suspensions		zeolite NaA suspensions with PVP	
ethanol:water (wt %)	zeta potential (mV)	suspension pH	zeta potential (mV)	suspension pH
0:100	–49.7 ± 0.3	10.3	–46.7 ± 0.9	9.8
30:70	–42.4 ± 0.4	10.3	–39.4 ± 0.2	10.4
40:60	–34.5 ± 0.3	10.4	–32.6 ± 0.3	10.5
50:50	–22.8 ± 0.4	10.2	–15.6 ± 0.3	9.6
100:0	–18.8. ± 0.6		–4.6 ± 0.2	

**Figure 4:**
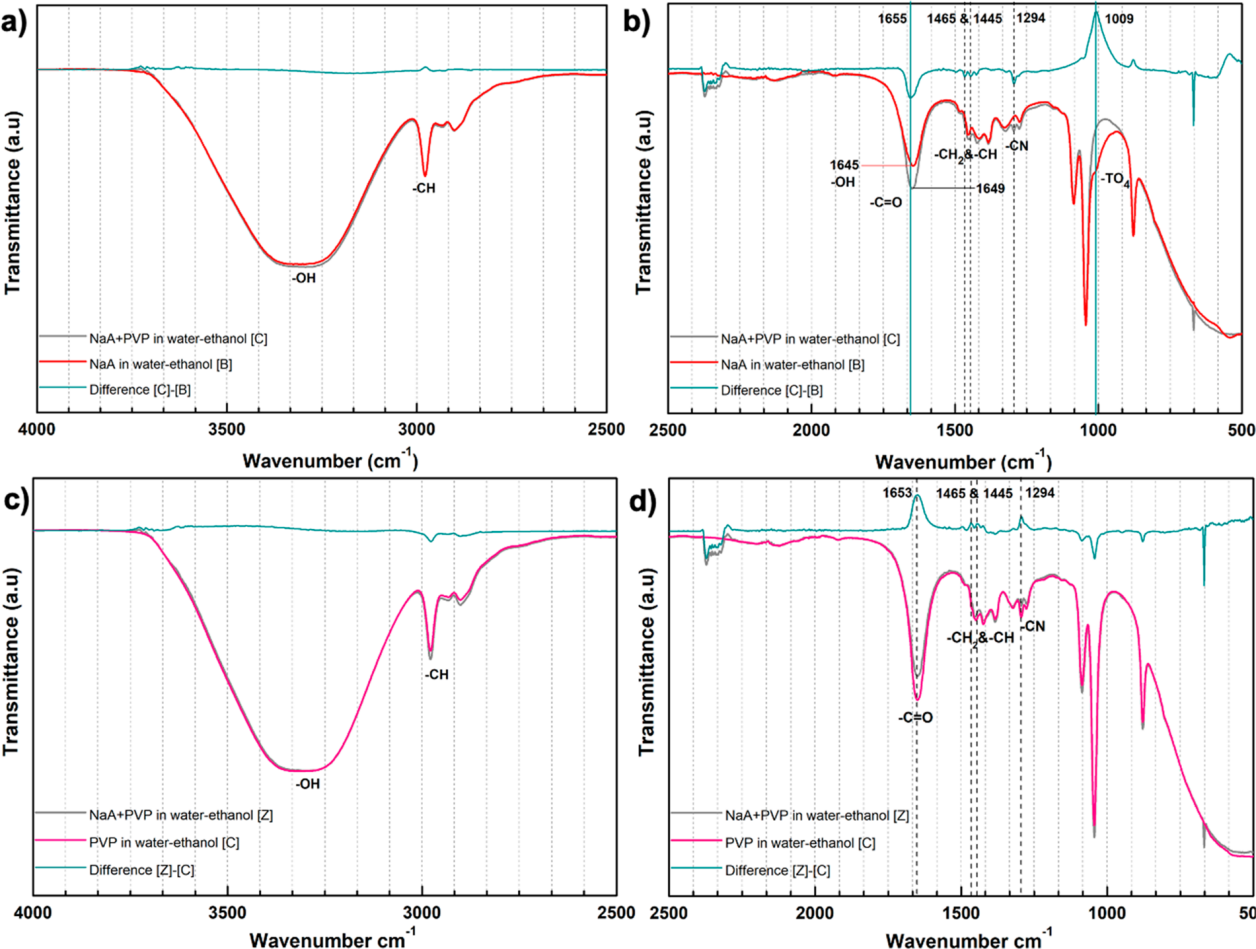
ATR-IR spectrum of zeolite NaA suspensions with (red spectrum) and without polyvinylpyrrolidone (PVP) (gray spectrum). Green spectra are for the difference of the gray and red spectrum. Samples contain 1.5 wt % zeolite-A with respect to suspension and 50:50 wt % ethanol:water. The amount of PVP addition is 10 wt % of the zeolite powder. Spectra are divided into two parts as the wavenumber range of 4000–2500 cm^–1^ (a) and 2500–500 cm^–1^ (b). The effect of zeolite addition into PVP-containing ethanol–water suspension is presented in (c) and (d).

To investigate chemical features of the interactions between the zeolite, the solvents, and PVP, ATR-IR analysis was employed. As shown in Figure 4a and b, the most obvious change occurring with the addition of PVP was detected in the difference spectrum. The positive difference at 1655 cm^–1^ was ascribed to the – C=O groups of the PVP molecules (further supported by analysis of Figurec S5c,d and 4d). The absorbance at 1645 cm^–1^ was assigned to the bending mode of hydroxyls in hydrated zeolite A,^72−76^ because it was observed for zeolite NaA in pure water or in the water–ethanol mixture (Figures 4a and S4a, respectively) but was absent in pure ethanol (Figure S5b). The negative band observed at 1009 cm^–1^ in Figure 4b after the addition of PVP is close to the bands of the zeolite, which are ascribed to the stretching vibrations of bridge bonds in TO_4_ (T: Si or Al) in the literature.^77^ The difference spectrum has a maximum that has a lower frequency than for the characteristic ones in zeolite NaA. We tentatively assign these negative bands to moieties that had been dissolved or been distorted by chemical interactions with the PVP.

### Stabilization of Zeolite NaA in Mixed Solvents

Colloidal stability of particles can be attained or improved in at least three ways: (i) Electrostatic stabilization, which relies on the electrostatic repulsion between particles due to their high zeta potential. This condition can be obtained in suspensions with low ionic strengths, and pH values far from the isoelectric point of zeolite powders. (ii) Steric stabilization is achieved by the coating of particles with polymer additives; thus, agglomeration of particles is prevented by the physical presence of additives between particles; (iii) Electrosteric stabilization in which the charged organic additives are used to ensure the dispersion of particles not only due to repulsive electrostatic interactions but also due to the physical presence of organic additives coating the particles. The applications of these three stabilization mechanisms in mixed solvent conditions were investigated.

### Electrostatic Stabilization of Zeolite NaA Powders in Ethanol–Water Mixture

**Figure 5:**
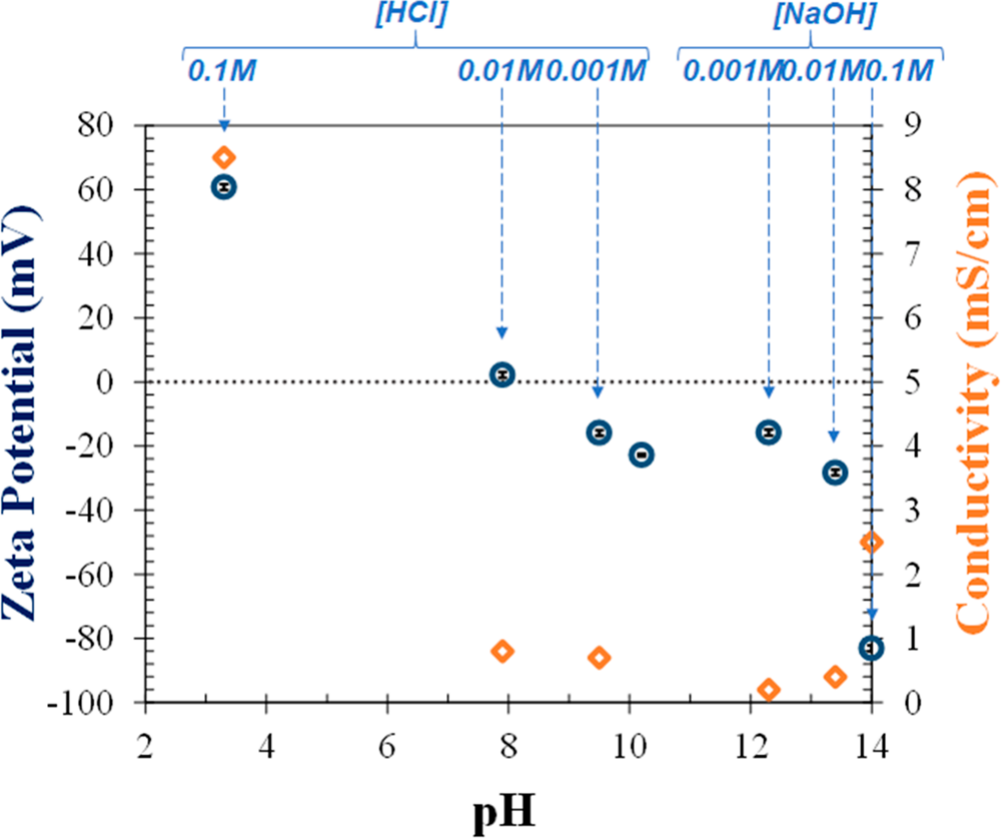
Zeta potentials of zeolite NaA in ethanol–water mixture (ethanol:water = 50:50) and solution conductivities as a function of pH. 0.001, 0.01, and 0.1 M HCl and NaOH were added to suspensions to change their pH.

For electrostatic stabilization of zeolite powders in the ethanol–water mixture, HCl and NaOH were added to the suspension as acid and base, respectively, to change the surface charge of the particles. Zeta potential values of the powders were presented as a function of the resulting pH in Figure 5. With the addition of 0.1 M HCl (pH: 3.3, zeta potential: −60.8 ± 0.9 mV) or 0.1 M NaOH (pH: 14.0, zeta potential: −83.1 ± 0.9 mV), the threshold value of ±30 mV required for electrostatic stabilization can be exceeded; however, in these measurements, the conductivity values increased significantly which may indicate the dissolution of zeolites. Therefore, changing the suspension pH for the electrostatic stabilization of zeolites in ethanol–water mixtures was not practical.

### Effects of Organic Additives on the Stabilization of Zeolite NaA Powders in Ethanol–Water Mixed Solvent

**Figure 6:**
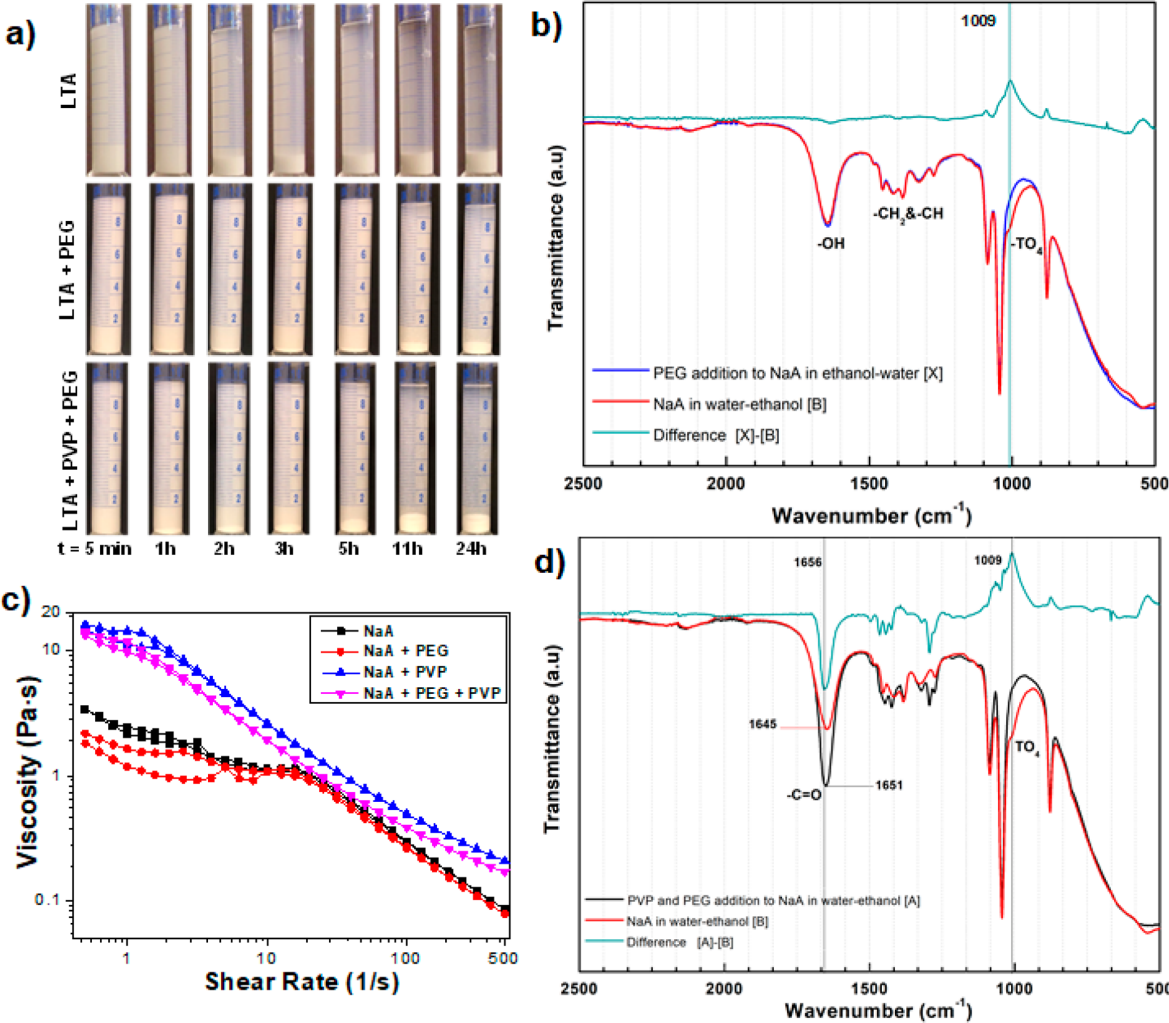
Effects of PEG addition (1 wt %) on zeolite-NaA dispersed in an ethanol–water mixture (50:50) with or without high molecular weight polyvinylpyrrolidone (PVP) addition of 10 wt %: sedimentation behavior (a), ATR-IR spectra of zeolite suspensions with (d) or without PVP (b), and rheological measurements of the suspensions (c). The green ATR-IR spectrum is the difference between the blue and the red spectra.

To stabilize the zeolite-NaA suspensions sterically or electrosterically, the effect of five organic additives, namely, PAA, PVP, PEG, CTAB, and SDS (chemical structures in Table S1), were studied (Figure S6). Among them, the additives with anionic character, PAA and SDS, sped up the sedimentation of zeolite particles as expected from the negative surface charge of the particles. On the contrary, the cationic surfactant, CTAB, slowed down the sedimentation of zeolite particles, but the effect was mild. The EDX analysis of the dried powders collected after sedimentation tests shows that charged species, such as the added polyelectrolytes, may also change the ion balance (Si/Al/Na ratios) of the zeolites (Table S2), which indicates the partial dissolution of the zeolites in use.^78,79^ Low molecular weight PVP addition resulted in the sedimentation of all particles in almost 2 h (Figure S6), while PEG addition could postpone this up to 11 h (Figure 6a).

To reveal the stabilization effect of the PEG addition on the ethanol–water suspension of zeolite NaA, the chemical and surface interactions between the PEG and the zeolite particles were investigated. As expected from PEG being a nonionic molecule, the pH of the zeolite suspensions stayed almost constant (Table 2). However, the zeta potential value changed significantly from −22.8 to −42.9 mV, which is almost equal to the zeta potential value of zeolite NaA powders in pure water, indicating that PEG molecules interact with the surfaces of the zeolite NaA particles. For this purpose, the zeolites with adsorbed PEG were analyzed using IR spectroscopy (Figure 6b). In the spectra, the absorbances between 1600 and 1635 cm^–1^ are attributed to −OH stretching vibrations while the ones near 2973 cm^–1^ are assigned to C–H stretching vibrations and the ones at 1085 and 1045 cm^–1^ are assigned to C–O vibrations. All these bands show the presence of water, ethanol, and PEG in solution. The only change observed in the IR spectra is the band at a wavenumber of 1009 cm^–1^, which is related to the asymmetric stretching vibrations of bridge bonds in TO_4_ (T: Si or Al). For the case with PVP addition, the disappearance of this band showed a strong direct interaction of PEG molecules with the zeolite structures. The strong interaction was supported by the change in zeta potential values irrespective of the suspension pH. Analysis of EDX spectra showed that the Na/Si/Al ratios in the zeolite structure were preserved in the presence of PEG molecules. In conclusion, the PEG molecules associate strongly with the zeolite NaA surfaces, which leads to an increase of the zeta potential, resulting in improved stabilization of the zeolite powders in the ethanol–water mixed solvent.

**Table 2:** Effect of Polyethylene Glycol (PEG) Addition on the Zeta Potential Values of Zeolite NaA in Ethanol–Water (50:50 wt %) Mixtures and pH of the As-Prepared Suspensions with High Molecular Weight Polyvinyl Pyrrolidone (PVP)

sample	zeta potential (mV)	suspension pH
zeolite NaA	–22.8 ± 0.4	10.2
zeolite NaA + PEG	–42.9 ± 0.8	10.1
zeolite NaA + PVP	–15.6 ± 0.3	9.6
zeolite NaA + PEG + PVP	–33.1 ± 0.84	9.8

### The Effect of PEG on the Stabilization of Zeolite-NaA Powders in Ethanol–Water Mixtures in the Presence of PVP

As depicted from the IR analysis and the zeta potential measurements, the repulsive interactions between zeolite particles were weakened because of their interactions with PVP molecules. Therefore, to obtain a homogeneous dispersion of zeolite NaA particles in an ethanol–water mixed solvent, there was a need for a dispersant that introduces a stronger interaction with zeolite NaA particles and prevents them from agglomeration. For this reason, PEG was used. PEG molecules were expected to effectively interact with charged zeolite NaA particles through hydroxyl (OH) end groups and oxygen (O) atoms on their backbone while preventing bridging between the zeolite NaA particles due to its short chains.

With the addition of PVP, the zeolite-NaA particles sedimented in about 1 h (Figure 3) in ethanol–water mixtures with high content of ethanol (50 wt %). With the addition of only 1 wt % PEG, the sedimentation time could be significantly extended to more than 5 h (Figure 6a) and the zeta potential of the zeolite NaA suspension increased from −15.6 to −33.1 mV (Table 2). This result shows that, regardless of the presence of PVP, the PEG interacts with the NaA particles and improves the effective electrostatic repulsion between the zeolite NaA particles in the solvent mixture. Furthermore, IR analysis (Figure 6d) shows that the absorbance at 1656 cm^–1^ is more intense in the difference spectra compared to the one in Figure 4b, indicating that PEG limited the interactions of PVP with zeolites NaA. Moreover, the absorbance peak at 1009 cm^–1^, which is the low frequency side of stretching vibrations of bridge bonds in TO_4_ (T: Si or Al), is absent. This occurred for additions of PEG or PVP. It was concluded that the corresponding moieties had either dissolved or been changed in their nature on the polymer adsorption. As a result, both polymers seem to interact chemically with the zeolite NaA particles. This conclusion was also supported by the changes of the zeta potentials and the suspension pH. However, the strength or the number of interactions between the surfaces of the zeolite NaA particles and PVP seem to be decreased because of the PEG.

Even though the sedimentation measurements can be used to compare the stability of powder suspensions, it is not practical for highly loaded suspensions and/or for suspensions contaning large molecules such as high molecular weight PVP. In such cases, rheological measurements can be used. It is known that the lower suspension viscosities commonly indicate better-stabilized suspensions.^80^ For the rheological investigations in this study, the zeolite-A content of suspensions was increased to 30 wt % while keeping the additive ratios constant with respect to the zeolites. As seen in Figure 6c, PEG addition decreased the zeolite NaA suspension viscosity both in the absence or presence of PVP. Considering the plot is logarithmic, the viscosity reduction achieved in the PVP-containing zeolite suspension is even more significant. When the power law was applied to the PVP-containing zeolite suspensions (η = *K*γ̇̇^*n*–1^), the *K* value was decreased from 12.7 Pa·s (*R*^2^ = 0.959) to 9.9 Pa·s (*R*^2^ = 0.986) with PEG addition, corresponding to about 22.4% reduction in viscosity over the range of applied shear rate. A reduced viscosity is generally indicative of a more homogeneous dispersion of powders in suspensions.^80,81^ The power law index, on the other hand, stayed about the same (0.31) for both suspensions, indicating that the flow characters of both suspensions were similar. Here, η is the viscosity, γ̇ is the shear rate, *K* is the consistency coefficient, which is an indication of the extent of viscosity, and *n* is the power law index showing the level of shear thinning behavior such that *n* = 1 is for Newtonian behavior and *n* = 0 is for very shear thinning behavior.

**Figure 7:**
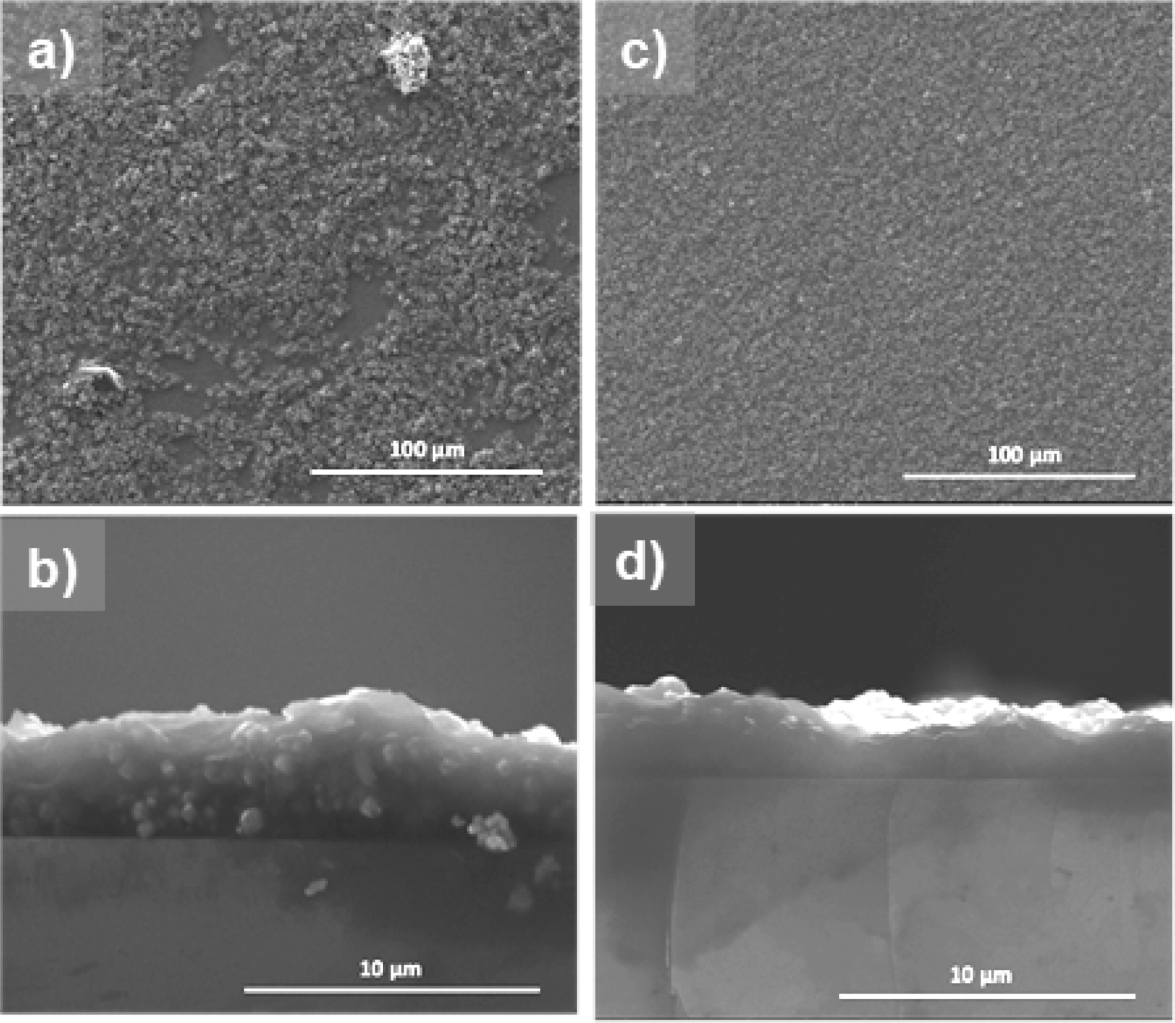
SEM micrographs of the zeolite NaA films obtained by spin coating of zeolite suspensions in PVP on silicon substrates with PEG addition (c, d) and without PEG addition (a, b). Top view on coatings in (a) and (c), cross sections in (b) and (d).

Finally, the effect of improvements in the dispersion of zeolite in water–ethanol suspensions by addition of PEG for shaping processes was demonstrated by spin-coating experiments on silicon wafers. Figure 7 shows that the zeolite suspensions dispersed with PEG resulted in a more homogeneous and continuous coating with lower roughness on the wafer compared to the less dispersed zeolite suspension in PVP.

## Conclusions

Many advanced shaping techniques, such as tape casting, spin coating, or electrospinning, require the use of organic solvents, such as ethanol, to dissolve organic additives and/or enhance the drying. Submicron-sized zeolite NaA particles can be easily dispersed in water due to their ionic nature as the zeolite powders typically have enough surface potential to exhibit electrostatic repulsion. However, this study showed that the repulsive energy barrier between the zeolite particles was significantly reduced in aqueous suspensions if ethanol was added at high amounts. In mixtures with 50 wt % ethanol, the zeta potential values of the zeolite-NaA particles decreased below the threshold value required for electrostatic stabilization, resulting in agglomeration and sedimentation of the zeolite particles. Ball milling and ultrasonic treatment were needed to break the agglomerated particles in the nanozeolite powder, and further measures were investigated to improve stabilization of the zeolite particles in ethanol–water mixtures. The addition of acid or base to increase the surface potential was found to not be a suitable method to achieve the required electrostatic stabilization of the zeolite-A particles in aqueous solvent mixtures with 50 wt % ethanol because it led to a partial dissolution of the zeolite. With the addition of cationic or anionic surfactants, further destabilization of the dispersions/suspensions was observed. Nonionic additives were identified as a suitable solution to be able to disperse zeolite-NaA in ethanol-rich aqueous solvent mixtures. Among them, the addition of 1 wt % PEG-400 led to zeta potential values of above −46 mV, indicating that sufficiently high electrostatic repulsion was achieved to stabilize zeolite-NaA for shaping in ethanol–water suspensions. The efficiency of PEG-400 as a dispersant was also reflected in reduced suspension viscosities and more uniform and continuous formation of zeolite layers in spin-coating experiments. This efficiency was also proven in the presence of PVP, which was chosen as a binder and slightly reduced the initial zeta potential (before addition of PEG). We believe that this approach is applicable for dispersing other types of low-silica zeolites in mixed alcohol–water mixtures and inspires further work for the shaping and use of these materials in various advanced shapes, such as coatings, nanofibers, or 3D printed structures.

## Supporting Information Available

Optimization of process parameters for ball milling and ultrasonication of as-received powders; XRD and CO_2_ isotherms of as-synthesized and treated zeolite NaA powder; FTIR analysis of zeolite A suspensions in pure water and in pure ethanol; the chemical structures of organic additives and their effect the sedimentation behavior and ion ratio of zeolite A (PDF)
