# Supplementary material for: Colloidal Stabilization of Submicron-Sized Zeolite NaA in Ethanol–Water Mixtures for Nanostructuring into Thin Films and Nanofibers
Source: Langmuir. 2022 Dec 20;39(1):192–203. doi: 10.1021/acs.langmuir.2c02241 (PMC9835981; doi:10.1021/acs.langmuir.2c02241)
Supplement: Supplementary file 1 — la2c02241_si_001.pdf [file la2c02241_si_001.pdf]

Supporting Information

Colloidal Stabilization of sub-micron-sized zeolite NaA in ethanol-water mixtures for nanostructuring into thin films and nanofibers

Oguz Gözcü[a], H. Utkucan Kayacı[a], Yibo Dou[c], Wenjing Zhang[b], Niklas Hedin[d], Alma B. Jasso-Salcedo[d,e], Andreas Kaiser[c], Simge Çınar Aygün[a,]*

[a]Dept of Metallurgical and Materials Engineering, Middle East Technical University (METU), 06800 Ankara, Türkiye.

[b]Dept. of Environmental Engineering, Technical University of Denmark, Bygninstorvet , 2800 Kongens Lyngby, Denmark

[c]Dept. of Energy Conversion and Storage, Technical University of Denmark, Anker Angelundsvej, 2800 Kongens Lyngby, Denmark.

[d]Dept. of Materials and Environmental Chemistry (MMK), Stockholm University, Svante Arrhenius väg 16 C, 10691 Stockholm, Sweden.

[e]Dept. of Biosciences and Agrotechnology, Centro de Investigación en Química Aplicada (CIQA), Blvd. Enrique Reyna Hermosillo 140, 25294, Saltillo, Coahuila, Mexico. (Present Address)

E-mail corresponding authors: * csimge@metu.edu.tr, Tel:+90-3122105937

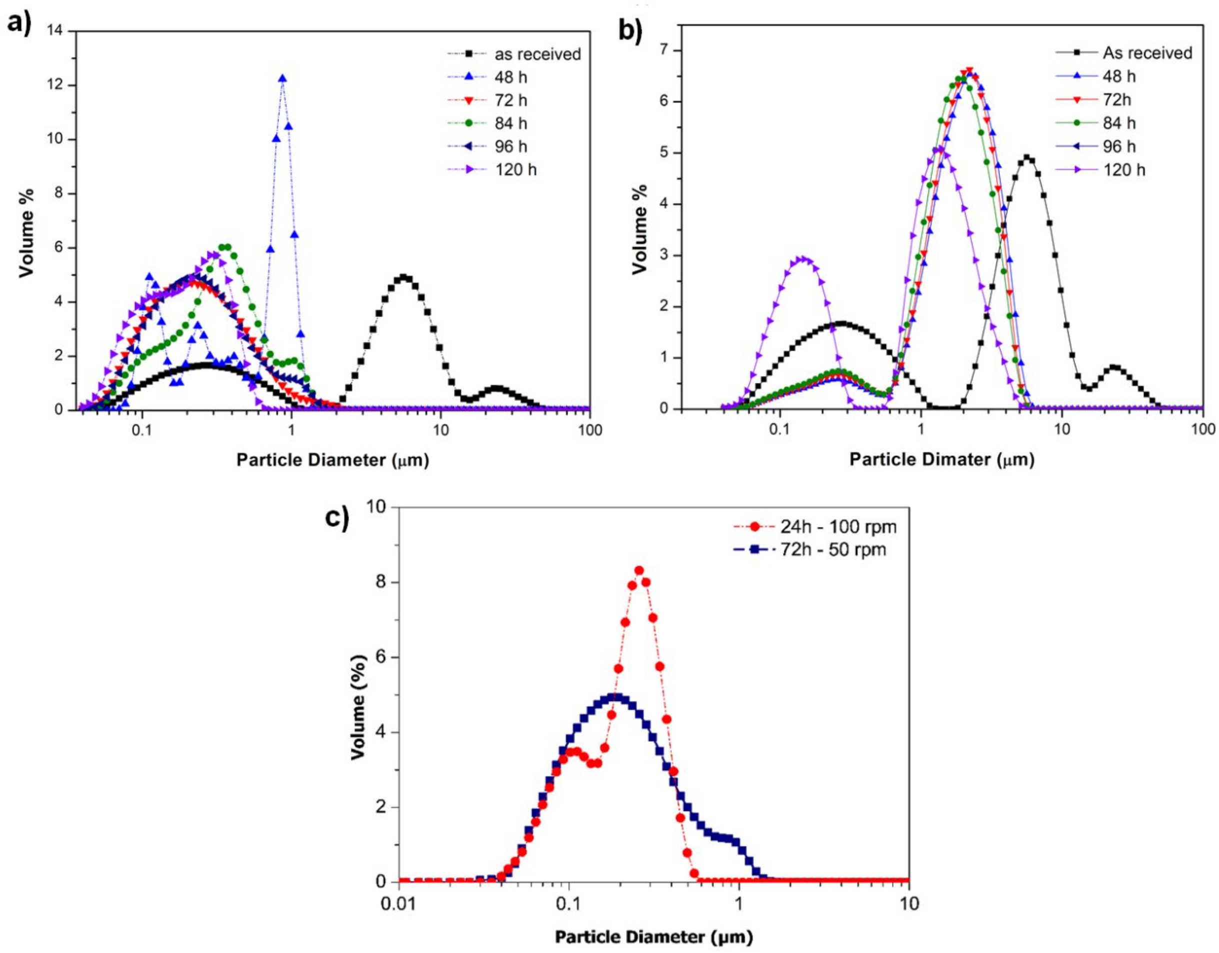

Figure S1: Optimization of process parameters for ball milling. Particle size distribution of zeolite A powders as a function of a) milling time at 50 rpm in water, b) milling time at 50 rpm in ethanol, and c) milling rate in water.

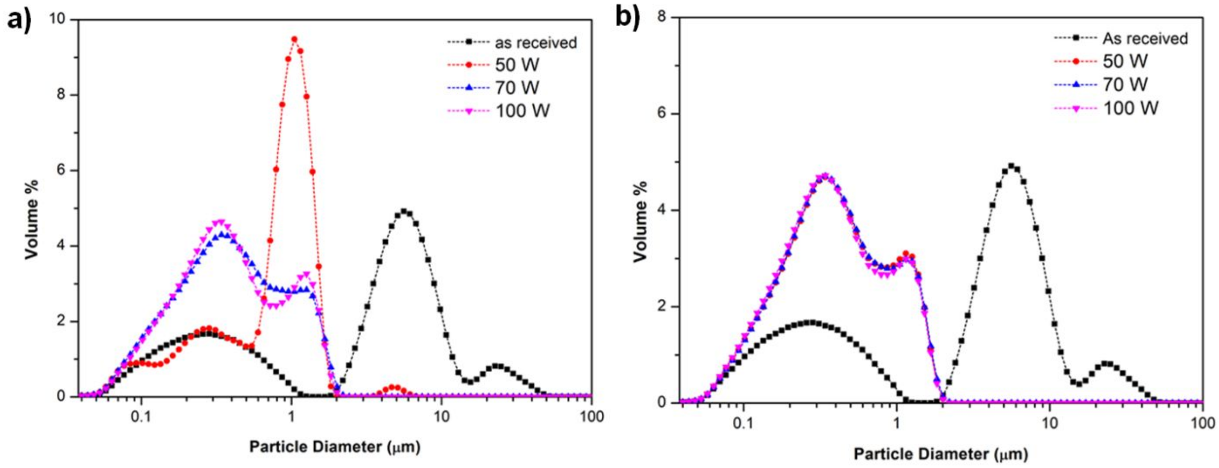

Figure S2: Optimization of process parameters for ultrasonication. Particle size distribution of zeolite A powder in water as a function of applied power for treatment duration of a) 3 minutes and b) for 5 minutes.

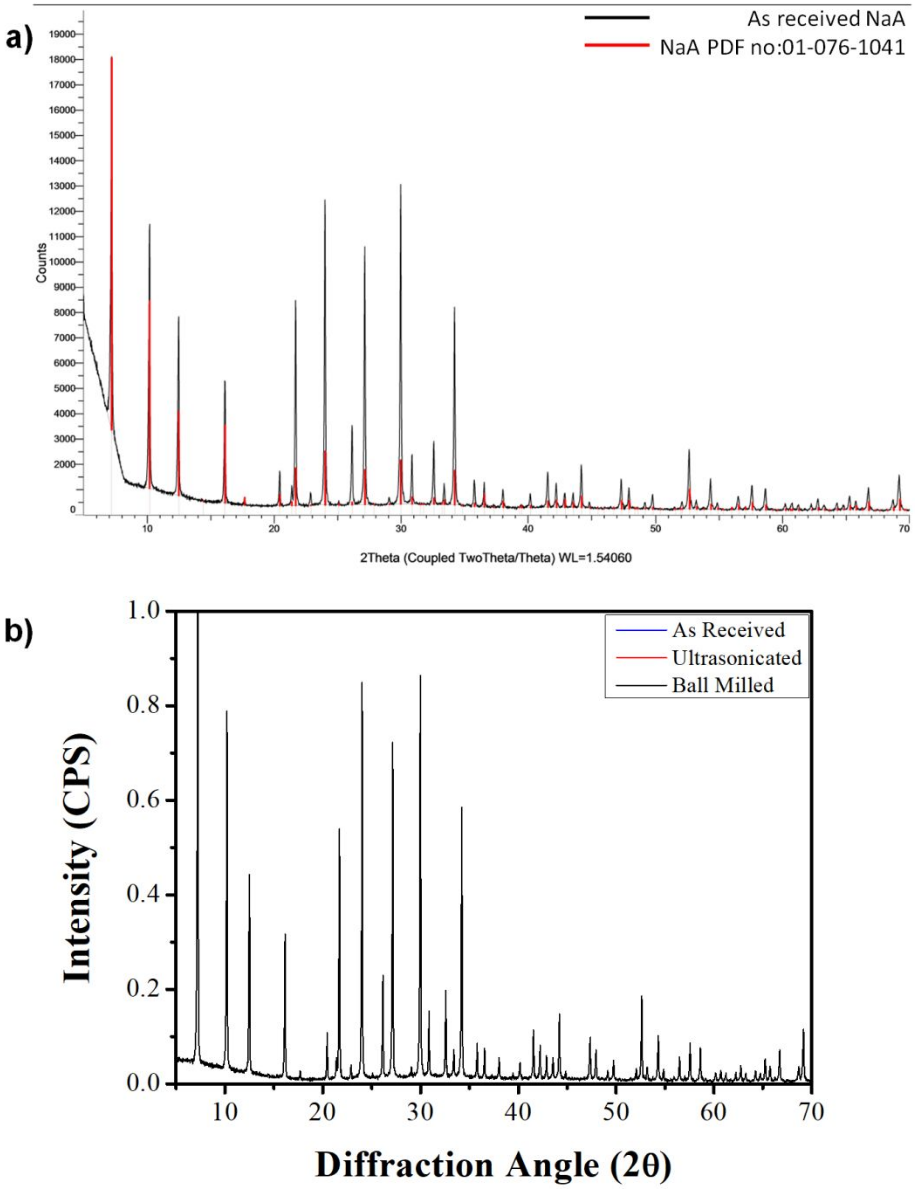

Figure S3: a) XRD Pattern of as-received zeolite NaA. This pattern coincides with XRD pattern with the PDF No: 01-076-1041. b) The influence of ultrasonication and ball-milling on the crystallinity of the zeolite A. All diffractograms are on top of each other.

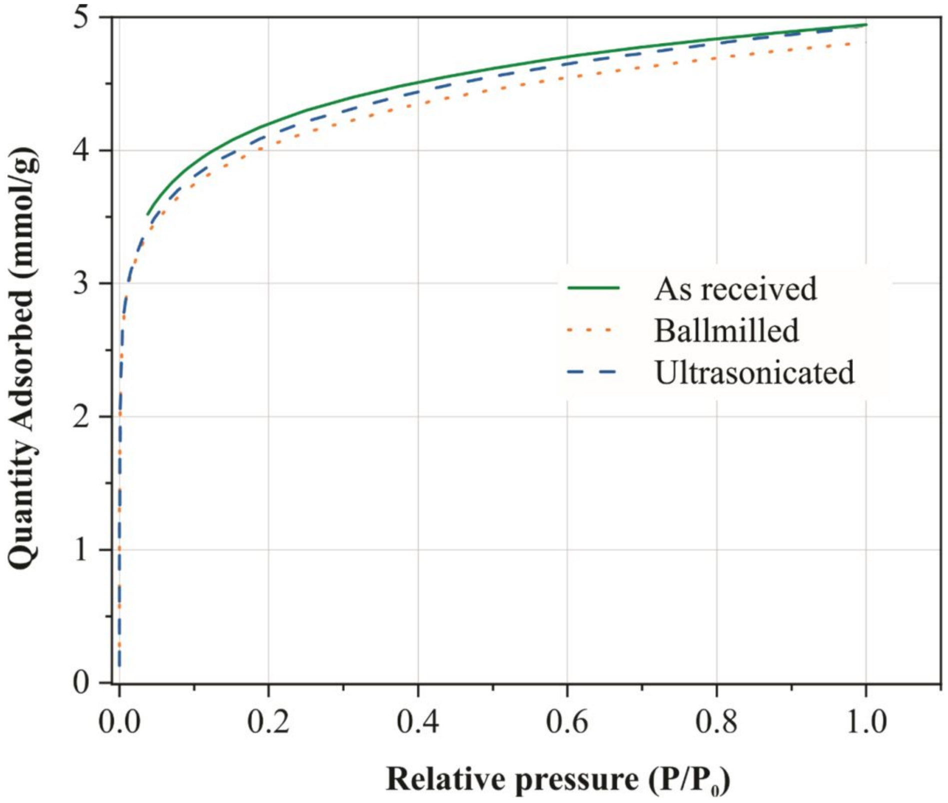

Figure S4: CO_2 equilibrium isotherms of as-synthesized and treated zeolite Na-A powders. As-synthesized powder (green); powders after ball milling in water (orange); and powders after ultrasound treatment in water (blue).

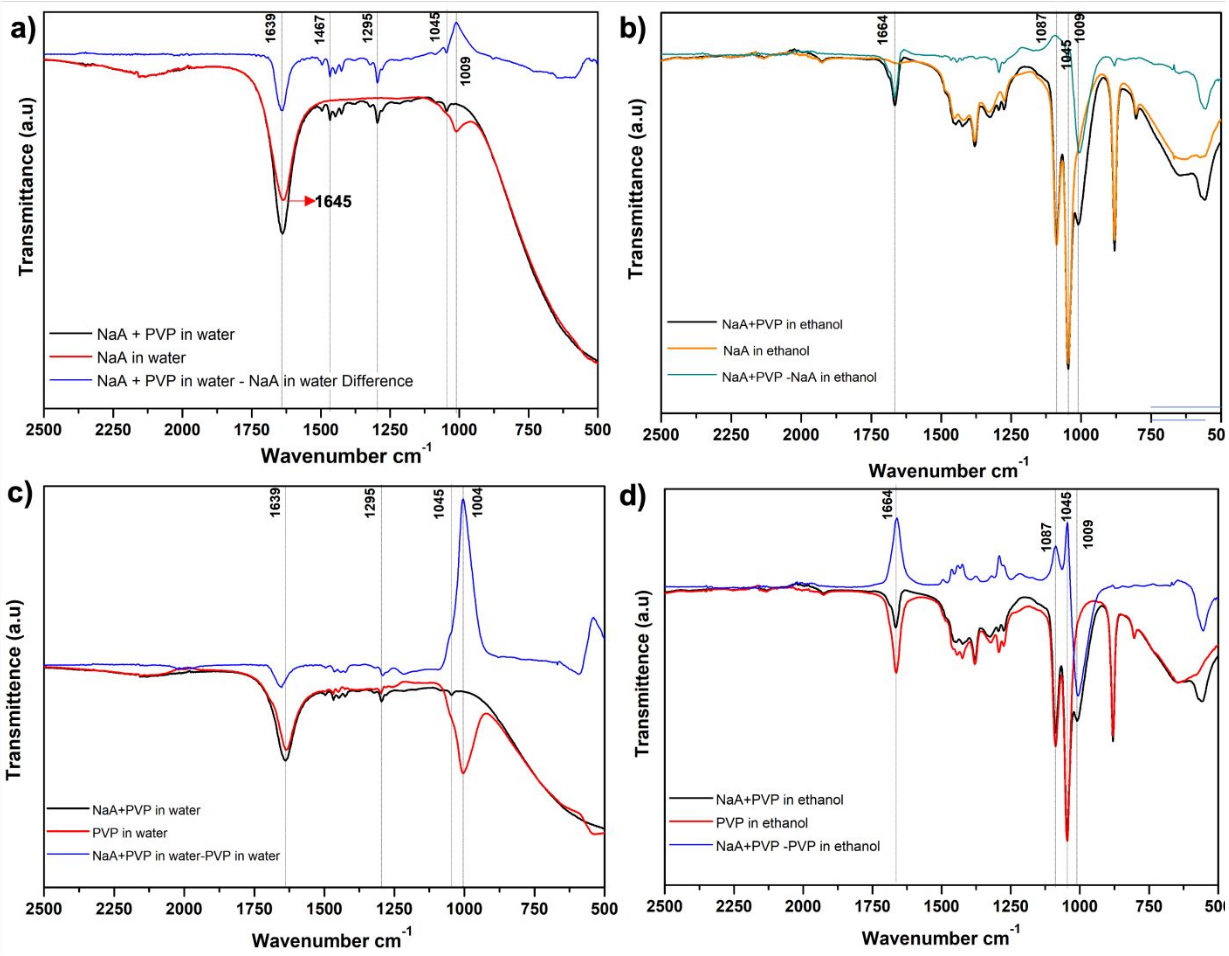

Figure S5: FTIR spectrum of zeolite A (called as NaA in the figure) suspensions in the absence and the presence of PVP and/or zeolite A in pure water (a and c) or in pure ethanol (b and d). Green spectra are for the difference of the gray and red spectrum. In order to distinguish the effects of water, ethanol\ zeolite and PVP, variations of spectra differences were employed (a vs c or b vs d).

Table S1: Chemical structures of additives

Additive	Chemical Formula	Chemical Structure
**Cetrimonium Bromide (CTAB)**	$C_{19}H_{42}BrN$	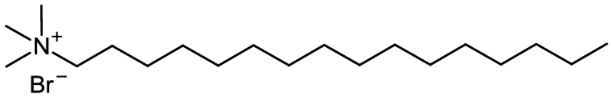
**Sodium Dodecyl Sulfate (SDS)**	$NaC_{12}H_{25}SO_4$	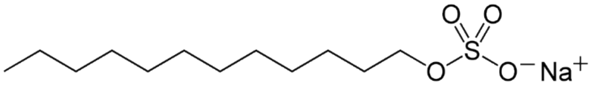
**Poly (acrylic acid) (PAA)** **MW: 5,000**	$(C_3H_4O_2)_n$	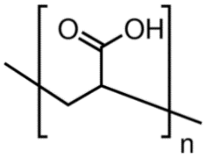
**Poly (ethylene glycol) (PEG)**	$(C_{2n}H_{4n+2}O_{n+1})$	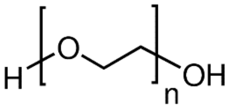
**Poly (vinyl pyrolidone) (PVP)** **MW: 1,500**	$(C_9H_9NO)_n$	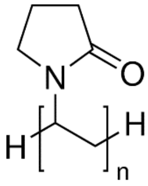

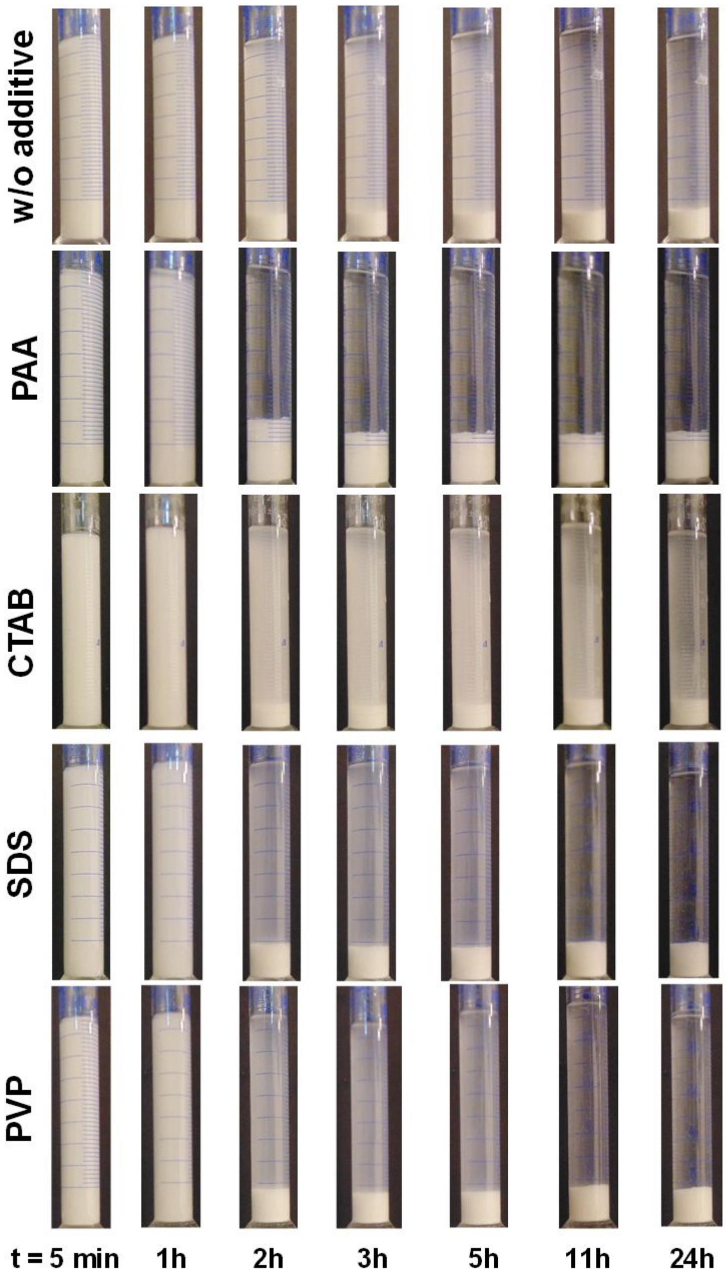

Figure S6: The effect of organic additives on the sedimentation behavior of zeolite A powders in ethanol-water solutions. a) the reference, without any additive; additions of b) PAA, c) CTAB, d) SDS and e) low molecular weight PVP (1,500). 1.5 wt% zeolite A powders used in ethanol-

water mixture (50:50 wt%) for all samples and polymer concentration was 1 wt% of the zeolite powder.

Table S2: Ion ratios in zeolite A powder dried after sedimentation experiments. Results were obtained using EDX Spectroscopy.

Additive	Na/Al ratio	Si/Al ratio
Without addition	0.53 ± 0.10	1.23 ± 0.22
PAA	0.54 ± 0.04	1.51 ± 0.17
CTAB	0.61 ± 0.15	1.29 ± 0.25
SDS	0.42 ± 0.10	1.34 ± 0.17
PEG	0.53 ± 0.11	1.24 ± 0.18
Low molecular weight PVP	0.55 ± 0.09	1.25 ± 0.10
